# Malaria Outbreak in Troops Returning from French Guiana

**DOI:** 10.3201/eid1211.060530

**Published:** 2006-11

**Authors:** Catherine Verret, Béatrice Cabianca, Rachel Haus-Cheymol, Jean-Jaques Lafille, Gisèle Loran-Haranqui, André Spiegel

**Affiliations:** * Ecole du Val-de-Grâce, Paris, France;; † Aquitaine Region of the French Police Force, Bordeaux, France

**Keywords:** Malaria, *Plasmodium vivax*, French Guiana, Outbreak, French troops, letter

To the Editor: In January 2005, the chief surgeon in a squadron of French policemen reported a cluster of Plasmodium vivax malaria attacks in troops returning from a 108-day operation in French Guiana. We conducted a retrospective cohort study to describe the malaria attacks and determine factors related to them.

A self-administered questionnaire was drawn up, with questions concerning operations in French Guiana (dates, locations) and preventive measures implemented against malaria. A malaria case was defined by the association of clinical signs and Plasmodium parasites in blood smears or quantitative buffy coat tests (per definition of military epidemiologic surveillance).

The 40-person mission in French Guiana (Operation Anaconda) took place from July 26, 2004, to November 6, 2004 (108 days of exposure). This mission against clandestine gold panning was conducted in a deep-forest environment where the troops were temporarily housed in villages of Brazilian gold panners. Occasionally, they washed themselves late in the evening in stagnant water near the river and patrolled outside during maximum biting periods. All troops received a chemoprophylaxis (doxycycline 100 mg daily) during the mission and for 4 weeks afterward.

From July 2004 through January 2005, 10 persons had >1 malaria attacks (attack rate 25%) for a total of 18 malaria attacks (incidence 13/100 person-months of exposure). P. vivax was isolated for 17 attacks and P. falciparum for 1 attack (Figure). Five patients had 1 malaria attack, and 4 patients had up to 3 relapses. Six patients had a malaria attack while receiving doxycycline.

**Figure:**
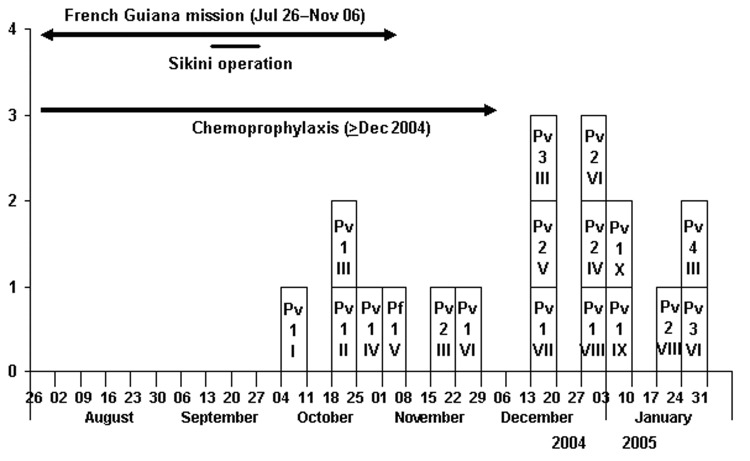
Epidemic curve of malaria attacks. Pv, *Plasmodium vivax*; Pf, *P. falciparum*; 1, access no.; I, case no.

Regarding chemoprophylaxis compliance, 34% reported missing <1 dose per week and 32% were fully compliant. The troops did not have permethrin-impregnated battlefield uniforms as do soldiers in the French Army. They had to impregnate their own uniforms with permethrin. Only 37% said they always wore clothing that fully covered them during the mission, and 86% reported having frequently used a repellent. All reported having slept under mosquito nets. No association was found between malaria attacks and regular chemoprophylaxis intake or use of repellents. Only 1 operation in French Guiana was associated with the risk of experiencing malaria attacks: 39% of troops located in Sikini had at least 1 malaria attack versus 7% of troops in other areas (relative risk: 5.9 [95% confidence interval 0.8–41.7]).

The incidence rate for this study was 10 times higher than the maximum incidence rate observed for French troops deployed in Côte d'Ivoire (1.3/100 troop-months in 2004). During an earlier Operation Anaconda, 37 of 62 persons deployed near the Sikini area had >1 malaria attacks (attack rate 61%). Of these, 30 had >1 attacks caused by P. vivax; occasionally an attack was associated with P. falciparum (*1*).

Our results suggest that the Sikini area was the high-risk area for malaria transmission (although the large confidence interval reflects a lack of power in our analysis). The operation dates (15–28 September) are compatible with the duration of the first cases of malaria occurrence.

French Guiana is the only French territory, except for Mayotte, where malaria is endemic, with nearly 5,000 cases per year, occurring mainly along the rivers bordering Suriname and Brazil (*2*). The highest frequencies of malaria appear during the dry season (September to December) in French Guiana (*3*), but no seasonality was described near the Brazilian border (*4*).

The Sikini area is located near the Oyapock River (Brazilian border). The mean annual incidence in Amerindians there is 48.6%, mainly due to P. falciparum (incidence 24.8%) and P. vivax (incidence 25.9%) (*2*).

P. vivax malaria incidence has increased in the Oyapock region, from 30% in 1987 to 50% in 2000–2004 (*2**,**4**–**7*). French troops were deployed in an area where parasite circulation was high. Troops had contacts with clandestine gold panners, mainly Brazilian illegal residents. This population, in which malaria incidence is almost impossible to evaluate, comes from Amapa State, where the incidence of malaria is increasing (*5*). In 2003, 60.9% of patients with malaria cases at Cayenne Hospital had a Brazilian name compared with 35.4% in 2000 (*6*). Also, the gold panners diverted the river and built basins where vectors could easily multiply (*7*).

Initial malaria attacks were treated with chloroquine or quinine. Five patients experienced >1 relapses (maximum 3 relapses). The relapses were treated with 50-mg daily doses of primaquine for 4 patients and by chloroquine for the fifth patient. Two patients had relapses after receiving primaquine. Primaquine resistance information was not available. However, resistance to primaquine has emerged in P. vivax strains (*8*).

We recommended that pre-impregnated battlefield uniforms be available for French policemen and chemoprophylaxis adherence be reinforced by directly observed intake by supervisory staff. Relapses of P. vivax malaria are a major therapeutic problem, particularly after primaquine therapy.
